# A Pilot Study Investigating the Effect of Music-Based Intervention on Depression and Anhedonia

**DOI:** 10.3389/fpsyg.2019.01038

**Published:** 2019-05-08

**Authors:** Thenille Braun Janzen, Maryam I. Al Shirawi, Susan Rotzinger, Sidney H. Kennedy, Lee Bartel

**Affiliations:** ^1^Music and Health Research Collaboratory, Faculty of Music, University of Toronto, Toronto, ON, Canada; ^2^Centre for Mental Health, University Health Network, Toronto, ON, Canada; ^3^Arthur Sommer Rotenberg Suicide and Depression Studies Unit, St. Michael’s Hospital, Toronto, ON, Canada; ^4^Faculty of Music, University of Toronto, Toronto, ON, Canada

**Keywords:** major depressive disorder, anhedonia, music-based intervention, music listening, rhythmic sensory stimulation

## Abstract

This study investigated the effect of a music-based intervention on depression and associated symptoms. Twenty individuals formally diagnosed with Major Depressive Disorder and in a current Major Depressive Episode (11 females and 8 males; aged between 26 and 65 years) undertook a 5 weeks intervention consisting of music listening combined with rhythmic sensory stimulation. Participants listened to a set of designed instrumental music tracks embedded with low-frequency sounds (30–70 Hz). The stimuli were delivered for 30 min, 5 times per week, using a portable consumer device with built-in stereo speakers and a low-frequency transducer, which allowed the low-frequency sounds embedded in the music to be experienced as a mild vibrotactile sensation around the lower back. Changes from baseline to post-intervention in measures of depression symptoms, sleep quality, quality of life, anhedonia, and music-reward processing were assessed with clinician-based assessments as well as self-reports and a monetary incentive behavioral task. The study results indicated that there were significant changes from baseline in measures of depression and associated symptoms, including sleep quality, quality of life, and anhedonia. However, individual differences in treatment response need to be considered. These findings corroborate previous evidence that music-based intervention, when added to standard care, is a promising adjunctive treatment for Major Depressive Disorder, and open new avenues to investigate the effect of music-based therapy to ameliorate anhedonia-specific dysfunction in major depressive disorder and other neuropsychiatric disorders.

## Introduction

Depressive disorders, including Major Depressive Disorder, are highly prevalent and disabling conditions with substantial personal and societal impact (Ferrari et al., 2013; Walker et al., 2015; World Health Organization, 2017). Major Depressive Disorder (MDD) is broadly characterized by persistent depressed mood and/or markedly diminished interest or pleasure accompanied by psychophysiological changes such as sleep disturbances, changes in appetite/weight, lethargy or fatigue, psychomotor agitation/retardation, and impaired cognitive function (American Psychiatric Association, 2013). This multifactorial and complex disorder is estimated to be the second leading cause of disability worldwide, affecting 4.4% of the world’s population (Seedat et al., 2009; Bromet et al., 2011; World Health Organization, 2017). Management primarily comprises psychotherapy and pharmacological treatment (Otte et al., 2016), however, common psychotropic medication (e.g., selective serotonin reuptake inhibitors) and adjunctive treatments often do not address reward-related symptoms in depression, such as anhedonia (Shelton and Tomarken, 2001; Nutt et al., 2007; Warden et al., 2007; McCabe et al., 2010).

Anhedonia is characterized by alterations in reward processing and is a core symptom of depression. There is mounting clinical, behavioral, and imaging evidence that individuals with depression who are anhedonic tend to respond differently than healthy individuals to primary and secondary rewards (for review, Sescousse et al., 2013; Admon and Pizzagalli, 2015; Lambert et al., 2018; Rizvi et al., 2018). This reduced reactivity to pleasant or rewarding stimuli arises from impairments in various components of reward processing, including interest/desire for reward, anticipation of reward, motivation and effort to attain reward, consummatory pleasure, and reward learning (Rizvi et al., 2016). Although recently emerging evidence suggests that distinct neuroanatomical areas underlie various facets of reward processing (e.g., Zhang et al., 2016), anhedonia is generally associated with reduced reactivity and connectivity of fronto-striatal circuits, including the prefrontal cortex (orbitofrontal, ventromedial prefrontal, and anterior cingulate), dorsal striatum (caudate and putamen), nucleus accumbens, and amygdala (Treadway and Zald, 2010; Der-Avakian and Markou, 2012; Pizzagalli, 2014; Admon and Pizzagalli, 2015). While there is substantial clinical and preclinical evidence that dopaminergic signaling in the mesolimbic circuit is crucial in reward processing, recent studies indicate that other neurotransmitters such as opioids, glutamate, gamma-aminobutyric acid (GABA), and serotonin also play a significant role and may be associated with different aspects of reward processing (Der-Avakian and Markou, 2012). Taking together, neuropsychological and neurobiological evidence collectively reveal a complex and multifaceted model of reward processing (Rizvi et al., 2016), which may underlie the relatively low response rates of common antidepressants and adjunctive treatments, prompting a need to investigate other avenues to ameliorate anhedonia symptoms in depression. In the present study, we explored whether music-based therapy may be such an approach.

Strong evidence from distinct lines of inquiry suggest that music has an important role in emotion evocation and hedonic regulation, and that such affective experiences are the main reasons for which people engage in musical activities in everyday life (Juslin and Laukka, 2004; Juslin and Västfjäll, 2008; Sloboda, 2010; Koelsch, 2014; Swaminathan and Schellenberg, 2015; Reybrouck and Eerola, 2017). There is considerable evidence that people actively listen to music to enhance positive emotions and regulate levels of arousal (Sloboda et al., 2001; Saarikallio and Erkkilä, 2007; Krause et al., 2014; Särkämö et al., 2014; Sakka and Juslin, 2018). Despite having no intrinsic biological or tangible value, pleasurable music experiences can elicit physiological, and autonomic responses (Blood and Zatorre, 2001; Menon and Levitin, 2005; Salimpoor et al., 2009), and strongly modulate activity in brain networks involved in emotion and reward processing, including cingulate cortex, orbitofrontal cortex, nucleus accumbens, and amygdala (Blood and Zatorre, 2001; Menon and Levitin, 2005; Koelsch et al., 2006; Salimpoor et al., 2013, 2015; Koelsch, 2014; Zatorre, 2015). Thus, the potential of music to modulate activity in brain circuits involved in reward, motivation, and emotion processing merits investigation for the treatment of MDD.

Music has been used in a variety of ways in the treatment of depression, including active music-making approaches – singing, playing or improvising with instruments (e.g., Koelsch et al., 2010; Erkkilä et al., 2011; Werner et al., 2017), and passive/receptive approaches, which generally involve music listening for relaxation, reflection, and mood regulation (for review, Maratos et al., 2008; Leubner and Hinterberger, 2017). Regardless of the approach, studies collectively report that music-based therapy, when added to standard care, significantly reduces depression, with particular effect on patients’ global state and functioning (Gold et al., 2009; Leubner and Hinterberger, 2017). However, despite growing evidence of the effect of music on depression, clinical evidence of the implications of music-based therapy on anhedonia-related symptoms is still sparse.

Therefore, the aim of the present study is to explore the effect of a music-based intervention combining music listening and low-frequency rhythmic sensory stimulation on depression and associated symptoms. While acoustic-driven stimulation of mechanoreceptors in the body with gamma-frequency sounds (30–120 Hz) has been long used as a means to reduce pain and to induce relaxation (Skille and Wigram, 1995; Punkanen and Ala-Ruona, 2012; Hollins et al., 2014), little is known regarding its effects on depression. Therefore, the first research question addressed in the present study is whether this music-based intervention would significantly reduce depression symptoms severity. Secondly, we were interested in the effect of the intervention on secondary measures such as sleep quality and quality of life, with particular attention to changes in anhedonia and music-reward processing. Finally, we also tested whether changes across time would interact with treatment response, thus revealing differences in response rate across our study sample.

## Materials and Methods

### Study Design and Ethics Statement

This open-label, single group, pilot study was a collaboration between the Music and Health Research Collaboratory/University of Toronto and the Canadian Biomarker Integration Network in Depression (CAN-BIND). All procedures were carried out in accordance with the recommendations of the University Health Network Ethical Guidelines of the University Health Network Research Ethics Board. Written informed consent was obtained from all participants in accordance with the Declaration of Helsinki. The study protocol was approved by the University Health Network Research Ethics Board (15-9799-AE) and registered at ClinicalTrials.Gov (NCT02685982).

### Participants

The study sample consisted of 20 outpatients between the ages of 18 and 70 years with a clinical diagnosis of MDD and who met the DSM-5 (American Psychiatric Association, 2013) criteria for a Major Depressive Episode, as determined by clinician assessment. At study enrollment, all participants were experiencing a depressive episode for ≥3 months, with a Montgomery-Asberg Depression Rating Scale (MADRS) (Montgomery and Asberg, 1979) score of ≥15, which indicates symptoms of at least mild to moderate intensity. Other inclusion criteria were self-reported satisfactory hearing bilaterally and English language fluency sufficient to complete the interviews and self-report questionnaires. Participants were included irrespective of medication status and were asked to continue their usual care during the study. Participants were excluded if they had any Axis I diagnosis (other than MDD) that was considered the primary diagnosis, had MDD with psychotic features, or had a diagnosis of Bipolar Disorder Type I or II. Presence of a significant Axis II diagnosis (borderline, antisocial) and a formal diagnosis of fibromyalgia was also exclusionary, along with high suicidal risk, substance dependence/abuse in the past 6 months, and presence of a significant neurological disorder, head trauma or other unstable medical condition. Participants who changed medication or adjusted medication dosage 4 weeks prior to enrollment, or that started psychological treatment during the 3 months prior to assessment were also ineligible for the study. Other exclusion criteria included: acute and active inflammatory conditions (e.g., rheumatoid arthritis, osteoarthritis, autoimmune disease); history of epilepsy or seizures; hearing impairment; pregnancy or breastfeeding; hemorrhaging or active bleeding; thrombosis or heart conditions such as hypotension, arrhythmia, pacemaker; prolapsed vertebral disc; and recent back or neck injuries.

### Procedures

Participants were recruited between March 2016 and August 2017 through clinician referrals from hospitals and health clinics of the Greater Toronto Area/Ontario/Canada. Study eligibility criteria were ascertained by the referring doctor and confirmed by the study psychiatrist during a screening visit. The screening assessment consisted of a comprehensive psychiatric evaluation to confirm the primary diagnosis comprising of MADRS, medical history, concomitant medication, and demographic assessments, and also included the completion of self-rated questionnaires such as the Hypomania Check-List (Angst et al., 2005) and the Revised Fibromyalgia Impact Questionnaire (Bennett et al., 2009).

A total of 23 patients were screened, of whom 3 were excluded due to a recent change in usual care (e.g., medication dosage) or substance abuse. Twenty participants were enrolled and provided written informed consent. One participant withdrew from the study and did not complete the post-intervention assessment, providing the final study sample of 19 patients (95% completion rate). Participants received 100 CAD$ at the end of the study in compensation for their participation.

The baseline visit was completed 1–7 days prior to the start of the intervention and included clinician-based assessments of depression symptoms severity and complementary demographic, and self-rated clinical measures of symptoms severity, sleep quality, quality of life, anhedonia, and music reward. The post-intervention visit was completed within 1–7 days after the completion of the last treatment session and assessed outcome changes from baseline. Additionally, participants completed a behavioral hedonic function task at both visits. Study compliance was assessed via phone or e-mail communication at weeks 2–4 of the intervention, as well as with the treatment logs submitted at the final visit.

### Intervention

The intervention consisted of music listening combined with rhythmic sensory stimulation.

In order to control for the rhythmic sensory stimuli administered, the music comprised of pre-recorded music tracks licensed from the Somerset SonicAid album “Music to Inspire Positive Thinking,” and consisted of slow tempo (50–80 BPM), instrumental, ambient music with embedded low-frequency sounds on the gamma-band range (30–70 Hz with particular emphasis on 40 Hz) and binaural detunement at 10–15 Hz. The music was delivered via a portable consumer device (Sound Oasis Vibroacoustic Therapy System VTS-1000; “Energize” track), which has a built-in low-frequency transducer located at the middle back region and two stereo speakers located at ear level. The low-frequency transducer allowed for the low-frequency sounds embedded in the music to be experienced as a mild vibrotactile sensation around the lower-back area as well as a low-level hum generated by the binaural detunement. Participants listened to the music for 30 min, 5 days per week, over 5 weeks, using the device which was provided to participants for the duration of the study. All 25 sessions were self-administered and completed at the patients’ homes. Participants were instructed to place the device on a comfortable chair or bed and enjoy the music. There were no restrictions regarding the type of activities participants could undertake during the 30 min session (e.g., reading, browsing the internet), however, participants were asked to complete a treatment log after each session containing information regarding the time and duration of the sessions, any activities performed during each session, as well as the device settings for the volume of the music and the intensity of the vibrotactile stimulation. The recommended intensity for the music and the vibrotactile stimulation corresponded to level 15 of the device (±5) but could be adjusted to a comfortable level if preferred. The treatment log also included a 7-point Likert scale where participants indicated how they felt immediately after the session in relation to the past 24 h. All patients received usual care during the study.

### Clinical Outcome Measures

The primary outcome measure of the study was the Montgomery–Asberg Depression Rating Scale (MADRS) (Montgomery and Asberg, 1979). This clinician-based measure consists of 10 items assessing symptoms such as sadness, inner tension, sleep disturbance, changes in appetite, anhedonia, pessimistic thoughts, and suicidal ideation. Each item is rated on a 6-point scale, with 0 being “normal/not present” and 6 being “extreme.” MADRS total scores range from 0 to 60, higher scores indicating more severe symptoms. This assessment was administered by the study psychiatrist or a trained researcher. Treatment response was defined as a 50% or greater decrease in MADRS score from baseline (Montgomery, 1994).

The secondary outcomes included self-rated measures of depression symptoms, sleep quality, quality of life, anhedonia, and music reward. The Quick Inventory of Depressive Symptomatology (QIDS SR-16) (Rush et al., 2003) is a self-report questionnaire comprised of 16 items assessing depression symptoms such as sleep disturbance, sad mood, changes in appetite/weight, concentration, self-criticism, interest, energy/fatigue, and psychomotor agitation/retardation. The total score ranges from 0 to 27, with higher scores indicating worse symptoms. The Pittsburgh Sleep Quality Index (PSQI) (Buysse et al., 1989) is a self-report questionnaire consisting of 19 items assessing sleep quality and disturbances over a 1 month period. The PSQI total score reflects seven different components (sleep quality, sleep latency, sleep duration, habitual sleep efficiency, sleep disturbances, use of medication, and daytime dysfunction), and ranges from 0 to 21, with higher scores indicating worse sleep quality. Quality of life was assessed using the Quality of Life Enjoyment and Satisfaction (Q-LES-Q Short Form) (Endicott et al., 1993), a self-report questionnaire with 16 items assessing domains such as physical health, leisure activities, social relationships, general activities, satisfaction with medications and life satisfaction. Each item is scored on a 5-point scale, with total scores ranging from 14 to 70, higher scores indicating better enjoyment and satisfaction with life (Stevanovic, 2011). The Snaith-Hamilton Pleasure Scale (SHAPS) (Snaith et al., 1995) is a 14-item self-report scale for assessing the hedonic capacity in patients with MDD and covers four domains of hedonic experience such as interests/pastimes, social interaction, sensory experience, and food/drink. SHAPS total scores range from 0 to 14, with a score above 3 indicative of anhedonia, and higher scores indicating higher levels of present anhedonia state. To evaluate how participants experienced reward and pleasure associated with music, we used the Barcelona Music Reward Questionnaire (BMRQ) (Mas-Herrero et al., 2013). This psychometric assessment tool consists of 20 items providing information on five distinct factors: musical seeking, emotion evocation, mood regulation, social reward, and sensory-motor. Each item is rated on a 5-point Likert scale (0: “completely disagree” and 5: “completely agree”) with a total score ranging from 0 to 100, higher scores indicating higher music-induced reward. Finally, daily ratings of the participant’s mood were recorded in the treatment log. Participants were asked to indicate on a 7-point Likert scale how they felt immediately after the session in relation to the past 24 h, where 1 indicated “very much worse” and 7 indicated “very much better.” Electroencephalographic (EEG) biomarkers were included as additional measures of the impact of the intervention on brain resting-state function and processing of rewarding information, but the analysis of these data will be reported separately.

Self-rated data were collected and managed using REDCap (Research Electronic Data Capture), an electronic data capture tool hosted at Brain-CODE (Harris et al., 2009). De-identified electronic data are stored in Brain-CODE (Ontario Brain Institute Centre for Ontario Data Exploration), a secure online neuroinformatic platform for data management, sharing, and analysis^1^.

### Monetary Incentive Task

Hedonic function was also assessed with a behavioral task featuring monetary incentives for successful trials (Knutson et al., 2000; Dillon et al., 2008). At the beginning of each trial, a visual cue is presented indicating the potential for monetary reward (+$) or no monetary incentive (0$). After a variable interstimulus interval (1–2.5 s, with 150 ms increments), a red target square is briefly presented on the center of the computer screen. The participants’ task is to press a computer key as fast as possible while the target is still on the screen. Visual feedback presented after each trial indicates whether the participant has successfully “hit” the target, for which he/she receives 50 cents, or whether the participant has pressed the button too early or too late. For trials with no monetary incentive, participants are given visual feedback about their performance and are made aware that successful trials will not be rewarded. No feedback concerning cumulative earnings is provided. Trials are separated by a variable interval ranging from 1.5 to 3 s, with 150 ms increments. The task involves eight blocks with 13 trials each, 104 trials in total. Blocks are separated by a 30 s break, and a longer break (1 min) is given after half of the blocks are completed. A fully balanced designed was used, with half of the total trials resulting in monetary reward and half no-incentive. A randomized stimulus presentation order was used within each block. With breaks, the task took approximately 15 min to be completed.

Before the task, participants received verbal and written instructions, and it was emphasized that the total amount of accumulated money (up to 26 CAD$) would be handed out at the end of the experiment. Participants were informed that not all correct responses would result in a monetary reward. The task was presented on a 24″ flat screen using E-prime software (version 2.0; Psychology Software Tools Inc., Pittsburgh, PA, United States). EEG data were also recorded during the task, but the analysis of these data will be reported separately.

### Data Analysis

Data analysis was completed according to per-protocol analysis and only included the data collected from participants who completed both study visits (*n* = 19). The effect of the intervention on the outcome measures was analyzed using a repeated measures analysis of variance with time (baseline and post-intervention) and treatment response (responders and non-responders) as factors. Significant time × response interactions were followed up with specific paired *t*-test comparisons. For the behavioral monetary incentive task data, mean reaction time to the target was analyzed with a repeated measures analysis of variance as a function of incentive cue (monetary reward, and no-incentive), study visit (baseline and post-intervention), and treatment response (responders and non-responders). Significant interactions were further explored with paired *t*-tests. Analysis of the daily mood changes after each treatment session (self-reported in treatment logs) was performed by averaging the daily ratings to reflect changes on a weekly basis. Changes of average mood ratings over the course of the study were analyzed with a repeated measures ANOVA with time (weeks 1–5) and treatment response (responders vs. non-responders) as factors. Before conducting each analysis, preliminary checks of ANOVA assumptions including normality, linearity, homoscedasticity, and homogeneity of regression slopes were performed and the final model was deemed acceptable if no violation of assumptions was identified. The effect size was calculated from the partial eta squared and is presented as percentages. Partial eta squared represents the amount of variance of the dependent variable (outcome) explained by the independent variable (intervention). Based on Cohen’s recommendations, a partial eta squared of 13.8% or greater was deemed a large effect size. Critical alpha was set to 0.05. All analyses were performed using IBM SPSS software version 22.0 (IBM Corp., Armonk, NY, United States).

## Results

### Baseline Characteristics

Data from 19 MDD patients were analyzed, and a summary of the demographic and clinical characteristics of the study sample at baseline is shown in Table 1. In total, 11 females and 8 males, aged between 26 and 65 years (*M* = 47.8, *SD* = 11.3) participated in the study. Of the total sample, 13 participants were on a regular psychiatric medication regimen and 6 were not taking medication as part of their standard care.

**Table 1:** Demographic and clinical characteristics of study participants at baseline.

	Responders (*n* = 7)	Non-responders (*n* = 12)	Total (*n* = 19)
Age (years)	49.5 ± 9.3	46.8 ± 12.6	47.8 ± 11.3
Sex (female/male)	5/2	6/6	11/8
Handedness (right/left)	7/0	10/2	17/2
Ethnicity (*N*)			
Asian Chinese	2	2	4
Latin American/Hispanic	2	0	2
Caucasian	2	9	11
Other	1	1	2
Marital status (*N*)			
Never married	0	6	6
Married/domestic partnership	2	5	7
Separated/Divorced	5	1	6
Education (*N*)			
High School/12th grade	2	4	6
College/No degree	2	1	3
College/University degree	3	7	10
Occupational status (*N*)			
Full-time employed	2	1	3
Unemployed, looking for work	4	2	6
Student	0	2	2
Keeping house	0	1	1
Disabled (permanent/temporary)	0	5	5
Retired	1	1	2
Psychiatric medication (yes/no)	5/2	8/4	13/6
MADRS total score (0–60)	30.71 ± 4.88	24.58 ± 5.16	26.84 ± 5.78

*Values are expressed as mean ± standard deviation or frequency.*

### Clinical Outcome Measures

A repeated measures ANOVA revealed a significant main effect of time in both the primary and secondary outcome measures, suggesting significant changes in measures of depression, sleep quality, quality of life, and anhedonia from baseline to post-intervention. A summary of the results is displayed in Table 2.

**Table 2:** Changes in outcome measures as a function of time and treatment response.

	Main effect of time			Time^∗^Response interaction			Responders (*n* = 7)		Non-responders (*n* = 12)	
	*F*	*p*	Partial η^2^	*F*	*P*	Partial η^2^	Baseline	Post-intervention	Baseline	Post-intervention
MADRS	109.398	<0.005	86.6%	66.771	<0.005	79.7%	30.71 ± 4.88	11.71 ± 4.66^∗∗^	24.58 ± 5.16	22.25 ± 7.23
QIDS	26.997	<0.005	61.4%	14.658	0.001	46.3%	16.71 ± 3.30	5.71 ± 3.63^∗∗^	11.83 ± 5.02	10.17 ± 3.95
PSQI	14.987	0.001	46.9%	5.917	0.026	25.8%	10.57±4.31	7.29 ± 5.79^∗^	10.17 ± 3.99	9.42 ± 3.29
QLES-Q	14.864	0.001	46.6%	12.421	0.003	42.2%	35.00 ± 8.85	48.00 ± 8.66^∗^	39.25 ± 5.39	39.83 ± 6.33
SHAPS	4.404	0.050	20.6%	8.134	0.011	32.4%	4.14 ± 2.85	0.86 ± 1.46^∗^	4.00 ± 2.86	4.50 ± 3.37
BMRQ total	1.768	0.201	09.4%	1.364	0.259	07.4%	70.29 ± 6.52	74.14 ± 10.15	64.42 ± 11.23	64.67 ± 5.75

*Main effect of time (baseline vs. post-intervention) and Time-by-Response interactions are reported, including partial eta-squared (η^2^) effect sizes. Paired sample t-test: ^∗∗^p < 0.005; ^∗^p < 0.05. Values are expressed as mean ± standard deviation. MADRS, Montgomery–Asberg Depression Rating Scale; QIDS, Quick Inventory of Depressive Symptomatology; PSQI, Pittsburgh Sleep Quality Index; QLES-Q, Quality of Life Enjoyment and Satisfaction; SHAPS, Snaith-Hamilton Pleasure Scale; BMRQ, Barcelona Music Reward Questionnaire total score.*

The analysis of the primary outcome measure (MADRS) showed a significant main effect of time [*F*_(1,17)_ = 109.398, *p* < 0.005] with reduction of depression symptoms from baseline to post-intervention (mean -10.667, SE 1.02, CI -12.818 to -8.515). There was also a significant time by response interaction (*F* = 66.771, *p* < 0.005), suggesting differences in treatment response within our study sample. Treatment response was determined by a minimum of 50% change in the MADRS total scores from baseline to post-intervention. Based on this criterion, 37% of participants (*n* = 7) presented a clinically relevant reduction of symptoms and were classified as responders (Table 2). The average improvement of depression symptoms for treatment responders was 62% (ranging from 50 to 86%), with mean MADRS score changing from 30.71 (*SD* = 4.88) at baseline to 11.71 (*SD* = 4.66) at post-intervention [*t*_(6)_ = 13.435, *p* < 0.005]. By definition, non-responders (*n* = 12) did not reach clinically significant changes in the primary outcome measure from baseline. As summarized in Table 2, there were no significant changes in all of the outcome measures for non-responders.

The self-rated measure of depression symptoms (QIDS) also presented a significant change over time (*F* = 26.997, *p* < 0.005, mean -6.333, SE 1.219, CI -8.905 to -3.762), and a significant interaction between time and response (*F* = 14.658, *p* < 0.005). The average decrease in symptoms for responders was 62%, improving from a baseline average score of 16.71 (*SD* = 3.30) to 5.71 (*SD* = 3.30) at post-intervention [*t*_(6)_ = 4.851, *p* = 0.003]. Moreover, 31.5% of participants reached an average score of ≤5 at post-intervention, which is considered as indicative of symptom remission (Trivedi et al., 2006).

There were also significant changes over time in measures of sleep quality (*F* = 14.987, *p* = 0.001, mean -2.018, SE.521, CI -3.118 to -0.918) and quality of life (*F* = 14.864, *p* = 0.001, mean 6.792, SE 1.762, CI 3.075-10.508). Significant time by response interactions suggested different response rates across participants in the study in relation to sleep (*p* = 0.026) and life satisfaction (*p* = 0.003). The average improvement in sleep quality for responders was 37% (Table 2). Importantly, 26.32% of responders reached a post-intervention PSQI score of 5 or less, which is accepted as indicative of a good sleeper with no insomnia (Buysse et al., 1989). In relation to quality of life, the average improvement for responders was 43%, changing from a mean Q-LES-Q score of 35 (*SD* = 8.85) at baseline to an average score of 48 (*SD* = 8.66) at post-intervention.

Significant changes in anhedonia symptoms from baseline to post-intervention (*F* = 4.404, *p* = 0.05, mean -1.393, SE.664, CI -2.793 to 0.007) and in time by response interaction (*F* = 8.134, *p* = 0.011) were also observed. Among responders, the average improvement of anhedonia symptoms was 80%, changing from a baseline average SHAPS score of 4.14 (*SD* = 2.85) to a post-intervention average score of 0.86 (*SD* = 1.46) [*t*_(6)_ = 2.860, *p* = 0.028]. According to established guidelines, a SHAPS total score of 4+ indicates significant impairment in hedonic capacity (Snaith et al., 1995). Based on this criterion, 11 of the 19 patients in our sample (58%) were anhedonic at baseline. At post-intervention assessment, 5 of the 11 anhedonic patients (45%) scored ≤ 3 in this questionnaire, suggesting a clinically relevant improvement in hedonic function. The analysis of the music-related reward assessment showed that there were no significant changes over time in the BMRQ overall scores and there was no significant time by response interactions. Although there was a numerical increase in the average BMRQ total score for responders from baseline (*M* = 70.29, *SD* = 6.52) to post-intervention (*M* = 74.14, *SD* = 10.15), this change did not reach statistical significance (*p* = 0.259).

The 7-point Likert scale in which participants recorded changes in mood after each treatment session was also analyzed. Daily mood ratings, as recorded in the treatment logs, were averaged to reflect changes on a weekly basis. Analysis indicated that there was a significant main effect of time (*F* = 7.72, *p* = 0.001, $\eta_{p}^{2}$ = 0.356) and treatment response (*F* = 7.22, *p* = 0.018, $\eta_{p}^{2}$ = 0.340), suggesting differences in mood rating from baseline between responders and non-responders. Further group comparisons showed that, during the first week of the intervention, there were no significant differences in mood ratings between responders (*M* = 5.1, *SD* = 0.73) and non-responders (*M* = 4.5, *SD* = 0.64, *p* = 0.114). However, as the intervention progressed, there were significant differences in the mood ratings after the sessions when comparing responders and non-responders (Table 3).

**Table 3:** Average mood ratings from weeks 1 to 5 between responders and non-responders.

	Responders (*n* = 7)	Non-responders (*n* = 12)	*p*
Week 1	5.1 ± 0.73	4.5 ± 0.64	0.114
Week 2	5.5 ± 0.79	4.5 ± 0.65	0.023
Week 3	5.7 ± 0.41	4.7 ± 0.76	0.016
Week 4	6.0 ± 0.80	4.7 ± 1.05	0.036
Week 5	6.2 ± 0.72	4.8 ± 0.97	0.011

*Values are expressed as mean ± standard deviation. Participants were asked to indicate on a 7-point Likert scale how they felt immediately after the session in relation to the past 24 h. Mood ratings ranged from 1 (very much worse) to 7 (very much better), with 4 indicating “no change.” Post hoc pairwise comparisons.*

### Monetary Incentive Task

**FIGURE 1:**
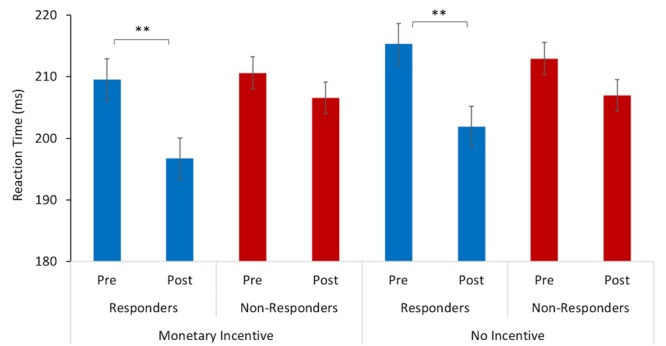
Reaction time to target stimulus as a function of incentive cue (monetary incentive, no-incentive). Changes in mean reaction time (ms) from baseline (pre-) to post-intervention for responders are represented with blue bars and for non-responders with red bars. The error bars represent standard errors. Statistically significant differences are marked with ^∗∗^ (*p* < 0.005).

A repeated measures ANOVA revealed a significant interaction between the mean reaction time and study visit (*F* = 18.50, *p* < 0.005, $\eta_{p}^{2}$ = 0.01), and an interaction between reaction time x study visit x treatment response (*F* = 3.70, *p* = 0.05, $\eta_{p}^{2}$ = 0.002). Pairwise comparisons indicated that there was a significant difference in the mean reaction time for responders between baseline (*M* = 209.55 ms, *SD* = 34.62) and post-intervention (*M* = 196 ms, *SD* = 37.85) for the trials where there was monetary incentive (*p* = 0.001). Similarly, responders also had faster reaction times at post-intervention (*M* = 201 ms, *SD* = 35.70) in relation to the baseline (*M* = 215.29 ms, *SD* = 39.73) in trials with no monetary incentive (*p* = 0.001). On the other hand, there were no significant changes for non-responders between baseline and post-intervention in trials with monetary incentive (*p* = 0.317) and no-incentive (*p* = 0.128), as displayed in Figure 1.

## Discussion

In this study, we examined whether a music-based intervention, which combined music listening and rhythmic sensory stimulation, would promote significant changes in depression and associated symptoms, including sleep quality, quality of life, anhedonia, and music-reward processing. Additionally, we assessed whether there would be differences in treatment response across our patient sample.

The study results indicated that there were significant changes in depression symptoms over 5 weeks of intervention. This finding is in line with previous systematic reviews and meta-analyses showing that adding a music-based intervention to standard care is a promising adjunctive treatment for depression (Maratos et al., 2008; Gold et al., 2009; Leubner and Hinterberger, 2017). However, we identified important differences in treatment response across our study sample. Using standard criteria for treatment response in clinical trials, 37% of patients in this study were responders and showed a clinically meaningful decrease in depression symptoms from baseline to post-intervention. The average improvement of symptoms for treatment responders was 62% (ranging from 50 to 86%), a result that was consistent using both clinician-based and self-report measures. Erkkilä et al. (2011) also reported differences in treatment response in their study, showing that 45% of depressed patients who undertook 3 months of active music therapy achieved a clinically relevant change in symptoms, with an average of 42.7% reduction in depression symptoms. The authors also reported the number-needed-to-treat, an epidemiological measure used to assess the effectiveness of a medication or treatment by estimating the number of patients who need to be treated in order to have a positive impact in one more individual. According to Erkkilä et al. (2011), for every four individuals to whom music-based therapy is offered, one person will change from no-response to response. This again demonstrates that there are individual differences in treatment response to music-based interventions for depression, pointing to the need to better elucidate possible factors underlying clinical responses in this population, such as depressive symptoms severity (Jenkins et al., 2018), individual differences in the reward value of music (Mas-Herrero et al., 2014; Martínez-Molina et al., 2016; Belfi et al., 2017; Loui et al., 2017; Mallik et al., 2017) and emotion-regulation strategies adopted during music listening (Carlson et al., 2015; Garrido and Schubert, 2015; Sakka and Juslin, 2018). In the present study, we attempted to control for some of these aspects by including patients with mild to moderate depression symptoms severity and assessing possible differences in how participants experienced reward and pleasure associated with music with the Barcelona Music Reward Questionnaire.

Results also indicated that there were significant changes over time in secondary outcomes, including sleep quality, quality of life, and anhedonia. Regarding sleep, findings suggested an average improvement of 37% in sleep quality among treatment responders. Additionally, it was observed that over 26% of patients who reported difficulties relating to sleep at baseline were considered good sleepers after 5 weeks. These results agree with recent studies suggesting that listening to music and music-based interventions may increase overall sleep quality (for review, De Niet et al., 2009; Wang et al., 2014; Jespersen et al., 2015; Feng et al., 2018), again speaking in favor of music-based therapy as an adjunctive treatment for depression, particularly when considering that common pharmacological treatments are often accompanied by adverse effects such as insomnia and sleep disturbances (Cassano and Fava, 2004; Otte et al., 2016). We also found significant changes in measures of quality of life from baseline to post-intervention, with responders reporting an average of 43% improvement in their perception of life satisfaction and enjoyment after the intervention. This finding is corroborated by research indicating that music activities (both music listening and music making) can significantly influence patients’ subjective views of their life satisfaction and enjoyment (Coffman, 2002; Grocke et al., 2009; Raglio et al., 2015).

There was also a marked change in anhedonia after 5 weeks. Self-report assessments indicated that there was an average of 80% improvement in anhedonia symptoms among participants who responded to the intervention. Moreover, 45% of patients who were anhedonic at baseline scored below the cut-off scores at the post-intervention assessment, suggesting a clinically relevant improvement in hedonic function particularly relating to consummatory pleasure (i.e., the hedonic response to rewards). These findings are complemented by the behavioral results of the monetary incentive task. The mean reaction time for responders in this task significantly improved from baseline to post-intervention, while there were no significant changes in reaction time for non-responders. Interestingly, responders were faster at both conditions – where correct responses resulted in a monetary reward and in trials with no monetary incentive. Considering that improvement in reaction time was observed only for responders, we cannot attribute these changes solely to a learning effect. Another point to note is that the monetary reward task adopted in this study did not include punishment trials, i.e., where participants need to respond rapidly not only to gain but also to avoid losing the monetary reward. This was done to control for anxiety-induced responses, particularly considering that patients with anxiety symptoms may be motivated by hypersensitivity to perceived failure (Treadway and Zald, 2010). Thus, one possibility is that these significant behavioral changes in treatment responders were indeed associated with improvements in the processing of anticipatory reward and motivation to pursue future rewards (Sherdell et al., 2011; Der-Avakian and Markou, 2012; Rizvi et al., 2016). Functional neuroimaging studies investigating responses to incentive cues indicate that impaired hedonic and reward-prediction processes associated with reduced activity in striatal regions, such as the nucleus accumbens and putamen (for review, see Pizzagalli, 2014), can be normalized with pharmacological treatment (Ossewaarde et al., 2011; Stoy et al., 2012) or psychotherapy (Dichter et al., 2009). The present results thus raise the possibility that music-based intervention may interact and improve circuit connections relating to reward processing in patients with depression and anhedonia.

There is robust evidence that pleasant music strongly modulates activity in fronto-striatal structures of the brain involved in reward/motivation and emotion processing (Salimpoor et al., 2013, 2015; Zatorre and Salimpoor, 2013; Koelsch, 2014; Mueller et al., 2015; Zatorre, 2015). For instance, it has been shown that reward anticipation during music listening increases dopamine availability in the dorsal striatum regions (Salimpoor et al., 2011), and that music reward value is directly linked to activity in mesolimbic striatal regions, especially the nucleus accumbens (Salimpoor et al., 2013). Recently, Mas-Herrero et al. (2017) modulated the activity of fronto-striatal pathways of the brain with transcranial magnetic stimulation during music listening and found that upregulation of the activity in this network increased perceived pleasure, psychophysiological measures of emotional arousal, and the monetary value participants assigned to the music. On the other hand, inhibiting the fronto-striatal circuit led to the opposite effects, decreasing perceived pleasure, arousal and motivation during music listening, thus reinforcing previous imaging evidence of the strong involvement of this network in processing reward, motivation, and emotion in music. Research has also shown that patients with depression who are anhedonic process and respond to music differently than healthy individuals possibly due to reduced activity in fronto-striatal regions of the brain (Osuch et al., 2009; Keller et al., 2013; Lepping et al., 2016; Jenkins et al., 2018). Therefore, it is possible that the participants who responded to the music-based intervention in this study were able to build a stimulus-reward association with the music resulting in significant improvements in depression and associated symptoms including anhedonia. Alternatively, treatment response could have been associated with music preference and how much participants experienced the pre-selected music as pleasant and rewarding. To confirm the role of music preference on treatment response, future research would benefit from assessing how much participants enjoyed the music listening experience.

Given that the intervention administered in this study combined music listening with rhythmic sensory stimulation, we also need to consider the effects of the gamma-band vibrotactile stimulation. Acoustic-driven stimulation of mechanoreceptors in the body by means of devices fitted with low-frequency transducers has been long used as a means to reduce pain and muscle tension, and to induce relaxation (Lundeberg et al., 1984; Zoppi et al., 1991; Kakigi and Shibasaki, 1992; Skille and Wigram, 1995; for review, see Punkanen and Ala-Ruona, 2012; Hollins et al., 2014). Studies have suggested that that local rhythmic vibratory stimulation resonates with mechanoreceptors in the body and modulates processes that occur at the spinal cord level inducing an effect known as vibratory analgesia (e.g., Hollins et al., 2003, 2017; Todd, 2010; Duan et al., 2014). Another potential mechanism of action of rhythmic sensory stimulation is neural entrainment. Research evidence indicates that the stimulation of sensory input pathways with rhythmic sensory stimuli may modulate brain oscillations (Thut et al., 2011). It has been repeatedly demonstrated that rhythmic visual stimuli (e.g., flickering light) (de Graaf et al., 2013; Spaak et al., 2014; Iaccarino et al., 2016; Notbohm et al., 2016), as well as auditory (Henry et al., 2014; Nozaradan, 2014; ten Oever et al., 2017) and somatosensory stimuli (Ross et al., 2013), induce phase-locking of brain oscillations at the same frequency of the stimuli. Thus, it is possible that the rhythmic sensory stimuli administered concomitantly with music listening may have induced changes in brain oscillations, serving as a potential underlying mechanism for the effects here reported. Indeed, there is evidence that depression is associated with abnormal neural activity (Linkenkaer-Hansen, 2005; Stewart et al., 2011; Olbrich and Arns, 2013; Fingelkurts and Fingelkurts, 2015; Leuchter et al., 2015), including frontal alpha asymmetry (Smit et al., 2007; Jaworska et al., 2012; Allen and Reznik, 2015; Baskaran et al., 2017) and thalamocortical dysrhythmia (Llinas et al., 1999; Schulman et al., 2011; Fuggetta and Noh, 2013; Ribary et al., 2014). This hypothesis warrants further investigation to examine whether rhythmic sensory stimulation with gamma-frequency vibroacoustic stimuli indeed drives brain oscillations and may serve to regulate dysrhythmias and enhance connectivity in depression.

Future studies are needed to confirm the present results. One of the limitations of the current design is the absence of a control condition, thus, it is not possible to rule out changes due merely to the passing of time, placebo, or to a Hawthorne effect. Another limitation to consider is that, at present, we are not able to dissociate the effects of the music listening component of the intervention from the influence of the rhythmic sensory stimulation on the results reported. Further randomized controlled trials are needed to better understand the distinct role of music listening and rhythmic sensory stimulation on depression and associated symptoms. Thirdly, we can also not exclude the influence of extraneous activities of participants during the treatment sessions and study duration. We attempted to account for the possibility that participants would engage in other activities during the self-guided sessions by asking them to record these activities in the treatment log (e.g., browsing the internet, reading, meditation). However, the lack of consistency in the recording of the data prevented an analysis of the type and frequency of these activities, and whether they could have interacted with the effectiveness of the intervention. Another limitation in this regard is that we did not control or record the emotion-regulation strategies adopted by each participant during music listening (e.g., distraction, rumination, reflection, discharge). Based on recent studies suggesting that people may engage in maladaptive or adaptive strategies during music listening, this factor could have influenced the present results (e.g., Carlson et al., 2015; Garrido and Schubert, 2015; Sakka and Juslin, 2018). We also acknowledge that the large number of outcomes in relation to the sample size may have imposed limitations in relation to statistical power. Finally, it is also possible that using researcher-designed music instead of self-selected music could have interacted with treatment response due to music preferences and motivation to complete the protocol, a point that should be considered in future studies.

## Conclusion

This pilot study suggests that a music-based intervention combining music listening and rhythmic sensory stimulation may improve depression and associated symptoms in patients with major depressive disorder. Of particular relevance is the finding that music-based therapy may be effective to ameliorate anhedonia-specific dysfunction in major depressive disorder and other neuropsychiatric disorders. These results open new avenues to further investigate the effect of music-based approaches on depression and anhedonia and to better elucidate the determinants of individual differences in treatment responses to music-based intervention.

## Ethics Statement

This open-label, single group, pilot study was a collaboration between the Music and Health Research Collaboratory/University of Toronto and the Canadian Biomarker Integration Network in Depression (CAN-BIND). All procedures were approved by the University Health Network Research Ethics Board (15-9799-AE) and registered at ClinicalTrials.Gov (NCT02685982). Written informed consent was obtained from all participants in accordance with the Declaration of Helsinki.

## Author Contributions

TBJ, SR, SK, and LB conceived and designed the study. TBJ and MAS acquired and analyzed the data. TBJ, MAS, SR, SK, and LB interpreted the data and prepared the manuscript.

## Conflict of Interest Statement

LB has served as a paid scientific consultant to Somerset Group and Sound Oasis and receives limited royalties from the Somerset Group for the SonicAid series and from Sound Oasis on the sales of the Vibroacoustic Therapy System VTS-1000 device. To manage this potential conflict of interest, the author was not involved in patient recruitment, consenting, or data collection process, and had no direct role in the data analysis process. SK has been an advisor/consultant for Abbott, Alkermes, Allergan, BMS, Janssen, Lundbeck, Lundbeck Institute, Otsuka, Pfizer, Servier, Sunovion; and participated in clinical trials/studies for Abbott, BMS, Janssen, Pfizer, Servier; speaking engagements with the following organizations: BMS, Lundbeck, Pfizer, Servier, Xian-Janssen; and has received research support from OBI, CIHR, BMS, Brain Canada, Janssen, Lundbeck, ORF, Pfizer, Servier. The remaining authors declare that the research was conducted in the absence of any commercial or financial relationships that could be construed as a potential conflict of interest.

## Footnotes

1 https://www.braincode.ca/
